# Contribution of the Cerebellum in Cue-Dependent Force Changes During an Isometric Precision Grip Task

**DOI:** 10.1007/s12311-015-0707-3

**Published:** 2015-07-26

**Authors:** Dieter F. Kutz, Barbara C. Schmid, Tobias Meindl, Dagmar Timmann, Florian P. Kolb

**Affiliations:** Institute of Physiology, Department of Physiological Genomics, University of Munich, Pettenkoferstr. 12, 80336 Munich, Germany; Department of Neurology, University of Duisburg-Essen, Hufelandstr. 55, 45138 Essen, Germany

**Keywords:** Classical conditioning, Forces, Human, Prehension, Multi-digit grip, Cued motor task, Dexterity

## Abstract

The “raspberry task” represents a precision grip task that requires continuous adjustment of grip forces and pull forces. During this task, subjects use a specialised grip rod and have to increase the pull force linearly while the rod is locked. The positions of the fingers are unrestrained and freely selectable. From the finger positions and the geometry of the grip rod, a physical lever was derived which is a comprehensive measurement of the subject’s grip behaviour. In this study, the involvement of the cerebellum in establishing cued force changes (CFC) was examined. The auditory stimulus was associated with a motor behaviour that has to be readjusted during an ongoing movement that already started. Moreover, cerebellar involvement on grip behaviour was examined. The results show that patients presenting with degenerating cerebellar disease (CBL) were able to elicit CFC and were additionally able to optimise grip behaviour by minimising the lever. Comparison of the results of CBL with a control group of healthy subjects showed, however, that the CFC incidence was significantly lower and the reduction of the lever was less in CBL. Hence, the cerebellum is involved not only in the classical conditioning of reflexes but also in the association of sensory stimuli with complex changes in motor behaviour. Furthermore, the cerebellum is involved in the optimisation of grip behaviour during ongoing movements. Recent studies lead to the assumption that the cerebello-reticulo-spinal pathway might be important for the reduced optimisation of grip behaviour in CBL.

## Introduction

The involvement of the cerebellum as a putative structure for associative learning of motor actions and sensory stimuli has been studied extensively in humans using the method of classical conditioning, for review see [1]. Simple avoiding reflexes have been most frequently tested as for instance the eye-blink reflex [2–5] or the lower limb withdrawal reflex in both reclining subjects [6–9] and standing subjects [10–12]. Avoiding reactions involving larger and more complex behaviour like a postural reaction to prevent a fall have also been studied, e.g. [13, 14]. A recent study showed, for instance, that anticipatory force changes in a task comparable to picking raspberry can be cued by an auditory stimulus [15]. In this study, the stimulus was associated with a complex motor behaviour that had to be readjusted during an ongoing movement that had already started.

Grip behaviour in humans is studied often by employing a lifting or an unloading task. Typically, the lifting task is divided into three phases. First, the grip phase, when the hand reaches the object and the fingers make contact. In this phase, the grip aperture and the grip force at contact are determined only by the object’s size and the expected weight [16, 17]. The second phase, the load or lifting phase, starts when subjects begin to lift the object from its support until lift-off. During the load phase, peak load and grip force rates are predictive scaled according to the expected object weight and finger and hand muscles work under isometric conditions so that force control depends also on tactile signals. The duration of the load phase depends on the weight of the object [18, 19] and lasts for up to 800 ms for the first lift of a novel object with a weight of 1 kg [16]. Changing the weight of the object results in a variation of the duration of the load phase. In general, the heavier the object the longer time required to lift it. The third phase, the hold phase, starts with the lift-off of the object and subject’s holding of the object. Handled objects frequently tend to rotate due to the relation of the individual finger forces with respect to the object’s centre of mass [20, 21]. Subjects compensate for the initial rotation by changing their individual finger forces and positions. In general, during the load phase, individual finger forces increase conjointly. After the transition to the hold phase, individual finger forces must be programmed separately. Hence, a broad change in the motor program is required during this transition. The duration of the hold phase depends on the experimental constraints and lasts several seconds, e.g. [18, 22].

Unloading tasks are used to study the ability of humans to anticipate load force changes due to external or self-produced perturbations [23]. Anticipation is a fundamental characteristic of the human motor system and enables adequate adjustment of muscular activation, even before proprioceptive or kinaesthetic information is available. Generating such anticipatory adjustments requires prediction to counteract a perturbation [24]. In unloading tasks, an object is held with one hand and the grip force depends both on the weight of the object, i.e. load force, and the coefficient of friction of its surface. Then, the object is lifted, which unloads the holding hand, either self-induced with the subject’s other hand or by an external device, e.g. by a robot. In the first case, anticipatory adjustments can be produced on the basis of an efferent copy of the voluntary action [24]. In the second case, subjects produce force adjustments after the perturbation, e.g. [25]. Anticipatory adjustments are produced only when the robot-induced perturbation coincides with a cueing stimulus. In this case, the unloading task also tests the ability to predict the time of the anticipatory adjustment [23]. This method is thus comparable to classical conditioning for testing associative learning in reflex systems.

Recently, we have introduced a new experimental approach, termed the “raspberry task”, which combines the loading task and unloading tasks. In the raspberry task, the duration of the pull phase—the equivalent to the load phase—can be changed randomly from 1 to 5 s [26]. Subjects are required to grip a specialised rod equipped with a force-sensor array to measure grip force and position of individual fingers [27, 28]. After having established a firm grip, isometric conditions are fulfilled for finger and hand muscles. Subjects are then asked to pull the locked rod steadily with a linearly increasing pull force until, unpredictably, the rod is unlocked (UUR). After unlocking, the rod moves due to the subject’s initial pull force and pull force is unloaded. Subjects are asked to stop the pull movement of the rod immediately. The transition from the locked to the unlocked rod required changes from increasing pull force to an immediate and rapid decrease of the force. Hence, in this task, the UUR—acting as an unconditioned stimulus in protective reflex conditioning, e.g. eye blink conditioning, for review see [29]—provokes an unpredictable force change with respect to time. The experimental approach is similar to grasping a raspberry and is comparable to the period in a lifting task immediately before the lift-off. UUR trials are combined with a preceding cueing stimulus. Hence, in the raspberry task, grip force and pull force increase conjointly during the early part of the pull phase whereas anticipatory adjustments can be observed in the late part of the pull phase.

One aim of this study was to examine the involvement of the cerebellum in establishing cued force changes in the raspberry task. A second objective was to analyse whether subjects presenting with degenerative cerebellar disease were restricted in minimising torque losses. For this purpose, we studied the occurrence of a cued reaction in the raspberry task and the development of torque production during the trials in a group of eleven patients with cerebellar disease (CBL) and in a group of eleven sex-, age-, and handedness-matched control subjects (CTRL).

## Materials and Methods

### Participants

The study was performed with the permission of the ethics committee of the Ludwig-Maximilians-University of Munich (No. 354–06) and was carried out in accordance with *The Code of Ethics of the World Medical Association* (*Declaration of Helsinki*). All subjects gave written informed consent to participation in the experiments. A group of eleven right-handed patients presenting with degenerative cerebellar disease (7 females, 4 males, age 51.0 ± 12.6 years) and eleven healthy, sex-, age-, and handedness-matched control subjects (age 50.7 ± 12.9 years) participated in the study. Each patient was examined by an experienced neurologist (D.T.). Characteristics of all participants and the clinical scores (ICARS [30] and SARA [31]) for the patients are given in Table 1. Data from both hands of each participant were analysed. The data from one hand of one patient (CBL-7) could not be included in the analysis because the patient did not grip the rod with sufficient force to avoid sliding. Hence, isometric conditions were not fulfilled for this hand and these data were excluded from the analysis.

**Table 1:** Characteristics of the subjects participating in this study

Patients (CBL)						Control subjects (CTRL)		
	Sex	Age (years)	Diagnosis	ICARS-Score (max. 100)	SARA-Score (max. 40)		Sex	Age (years)
CBL-1	f	30.9	SAOA	39.5	12.5	CTRL-1	f	33.0
CBL-2	f	41.0	SAOA	43.0	15.5	CTRL-2	f	41.0
CBL-3	m	41.6	SAOA	19.0	8.0	CTRL-3	m	40.3
CBL-4	f	42.9	ADCA-III	25.0	9.0	CTRL-4	f	41.2
CBL-5	m	45.2	ADCA-III	30.5	10.0	CTRL-5	m	44.0
CBL-6	m	46.2	SAOA	33.5	13.5	CTRL-6	m	45.1
CBL-7	m	55.2	SAOA	18.5	n.a.	CTRL-7	m	55.0
CBL-8	f	55.2	ADCA-III	17.0	5.5	CTRL-8	f	53.7
CBL-9	f	62.6	SAOA	18.5	9.5	CTRL-9	f	62.7
CBL-10	f	69.8	SAOA	27.0	10.0	CTRL-10	f	70.9
CBL-11	f	69.9	SCA-6	38.5	13.0	CTRL-11	f	70.9
Total	7f/4m						7f/4m	
Mean ± SD		51.0 ± 12.6		28.2 ± 9.5	10.7 ± 3.0			50.7 ± 12.9

All subjects were right handed

*f* female, *m* male, *ADCA-III* autosomal dominant cerebellar ataxia of type III, *SAOA* sporadic adult onset ataxia, *SCA-6* spino-cerebellar ataxia of type 6, *ICARS-Score* International Cooperative Ataxia Rating Scale for pharmacological assessment of the cerebellar syndrome [30], *SARA-Score* Scale for the Assessment and Rating of Ataxia [31], *n.a.* not available

### Force-Measuring Film and Grip Rod

Subjects were asked to apply a pull force to a specialised grip rod equipped with a force-measuring film to measure grip force exerted by individual fingers, and a force transducer to measure the pull force. The grip rod (diameter 20 mm) and force-measuring film have been described elsewhere in detail [26, 28]. Briefly, the force-measuring film (type 3000/HOT, TekScan, MA, USA) allows the simultaneous recording of the grip force and position of individual fingers. It consists of a rectangular array of 180 resistor-based pressure sensors, covering an area of 61 × 75 mm with a sensor density of 4 cm^−2^. The force range of each single sensor is 0.9–13.3 N with a resolution adjusted to 0.1 N [26]. Sampling frequency for each sensor is 150 Hz.

The grip rod is driven by a linear motor (type: STA2505, Copley Controls, Canton, MA, USA) that moves the rod axially (i.e. horizontally and coronally with respect to the sitting subject) up to an amplitude of 100 mm with a maximum force of 25 N. The position of the rod is measured by a linear potentiometer (type: REM 13-200-K, Megatron Elektronik, Putzbrunn, Germany). The forces applied in axial direction (pull force) were recorded by a force transducer (type: U9B, Hottinger Baldwin Messtechnik, Darmstadt, Germany). The motor is controlled, and position and pull force are recorded using software custom-written in LabVIEW (v. 8.2, National Instruments, Austin, TX, USA). The sample frequency for the motor control was 100 kHz and for position- and pull-force recording 1 kHz.

### The Raspberry Task

The raspberry task is a precision grip task simulating the prehension involved in picking a raspberry, a procedure that requires continuous adjustments of grip and pull forces [27]. The task can be divided into three phases. In the grip phase, the subject applies the fingers to the rod. Then pull force should be increased linearly while the rod is locked (pull phase). The start of the pull phase (*t*_start_) is defined as the time at which the subject’s pull-force slope exceeded a threshold of 0.5 N/s. The pull phase lasts for a random time interval of 1–5 s until the rod is unlocked. The time of unlocking was unpredictable for the subject (unpredictable unlocking of the rod, UUR). UUR is comparable to the time at which the raspberry detaches from the bush. In terms of classical conditioning, UUR represents the unconditioned stimulus (US). Finally, after the rod is unlocked, subjects should diminish pull force rapidly (“pluck phase”).

All participants were instructed by the same words in German. The instruction was: “Grip the rod with three fingers. Pull the rod gently in the horizontal direction. Pull with linearly increasing force, as though you were plucking a raspberry from a bush. When the rod starts to move, stop it with as little effort as possible.” Subjects were alerted by a red light-emitting diode when the pull-force slope exceeded a given maximum value predetermined of 5.5 N/s. The threshold for patients was set to 7.5 N/s because they were often not able to produce pull-force slopes of lower values.

During the pull phase, neither fingers nor the arm moved, so that isometric conditions are established. Because pull force and grip force must be carefully coordinated, any increase of pull force had to be due to an increase in grip force. Hence, the analysis of grip force and pull force is restricted to the pull phase only.

### Experimental Procedure

The standard delay paradigm was used to study cue-dependent force changes comparable to classically conditioned reflex tasks according to the protocol suggested by Gormezano and Kehoe [32]. A time-locked sequence of a preceding cueing stimulus (CS) and the UUR, both co-terminating, was given. The CS consisted of an auditory signal (1000 Hz, 75 dB, duration: 465 ms) given to the ear of subject’s dominant hand side via headphones. The CS was superimposed on a continuous pink noise of 50 dB SPL, applied bilaterally, to mask environmental noise. The CS-UUR window was fixed at 465 ms. No instructions were given to the subjects regarding the CS to obtain comparable conditions to classically conditioned tasks. The inter-trial intervals varied randomly from 10 to 15 s. Subjects were tested within a single session consisting of three consecutive sections for each hand. In the first section, 20 UUR-alone trials were carried out, during the second section, 50 paired trials with both CS and UUR were carried out, and during the last section, ten UUR-alone trials were carried out as a control (post UUR-alone trials). Prior to the first section, at least ten test trials were carried out to familiarise subjects with the perturbation and to reduce initial adaptation effects. After the first four blocks of ten paired trials, an UUR-alone trial was inserted after each block for comparison. After the last (fifth) block of paired trials, five CS-alone trials were then recorded at random inter-trial intervals to check for the extinction of the cued force change (CFC). Both hands of each subject were tested, with the non-dominant hand first. The results of both hands are treated as independent measures of the whole group and were analysed separately. Since the aim of the study was not related to handedness, the data from the dominant and non-dominant hands were pooled.

### Analysis of Pull-Force Slope Change

In a precision grip task, subjects exert sufficient grip force to prevent the object from slipping, while at the same time avoiding excessive grip forces [18, 23, 25, 33, 34]. To prevent slipping the condition grip force × friction coefficient > pull force must be fulfilled [35]. Any changes in the pull-force slope thus result from changes in the grip forces. Hence, pull-force slope changes are analysed for simplicity. In a preceding study, an algorithm for the detection of pull-force slope changes during the pull phase was described [35]. This algorithm is applied to detect pull-force slope changes due to associative learning of a cueing stimulus during the pull phase [15].

In general, the time of pull-force slope change (*t*_break_) is determined using a two-linear-splines regression model:

1 $$ \begin{array}{c}\hfill \mathrm{P}\mathrm{F}(t)=\left\{\begin{array}{c}\hfill \mathrm{pull}\kern0.5em \mathrm{force}\left({t}_{\mathrm{break}}\right)-{\beta}_1\left(t-{t}_{\mathrm{break}}\right)\kern1em for\kern0.5em t\le {t}_{\mathrm{break}}\hfill \\ {}\hfill \mathrm{pull}\kern0.5em \mathrm{force}\left({t}_{\mathrm{break}}\right)-{\beta}_2\left(t-{t}_{\mathrm{break}}\right)\kern1em for\kern0.5em t>{t}_{\mathrm{break}}\hfill \end{array}\right.\hfill \\ {}\hfill \mathrm{P}\mathrm{F}(t)=\mathrm{estimate}\kern0.5em \mathrm{of}\kern0.5em \mathrm{pull}\kern0.5em \mathrm{force}(t),\kern1em {\beta}_{1,2}=\mathrm{slopes}\hfill \end{array} $$

where the coefficients are estimated in a least-squares setting [35]. This algorithm is used to determine a pull-force change in the interval from *t*_start_ to CS (i.e. the equivalent time in UUR-alone trials) dividing this interval in two intervals: interval-1 [*t*_start_, *t*_break_] and interval-2 [*t*_break_, CS].

For classification of cued trials, the algorithm is extended for each subject separately as follows:

1. For all paired trials, the time of pull-force change is determined by Eq. 1 during the interval from CS to UUR. This time is called *t*_CSbreak_ and marks the onset of the cued response (CR) (Fig. 2c).
2. For all UUR-alone trials, the mean pull-force slope γ ± SD_γ_ is calculated for the interval [t*, UUR] with t* = UUR - 465 ms. Then the 95 %-confidence band (CB_0.95_) of the pull-force slope is calculated as follows:

2 $$ \begin{array}{l}{\mathrm{CB}}_{0.95}=\gamma \pm z\kern0.1em _{\mathrm{Bonferroni}}\times {\mathrm{SD}}_{\gamma}\\ {}\mathrm{with}\kern1em {z}_{\mathrm{Bonferroni}}={\varPhi}^{-1}\left(1-\frac{0.05}{N_{\mathrm{CSUS}}}\right)\\ {}\mathrm{and}\kern0.5em {\varPhi}^{-1}=\mathrm{inverse}\ \mathrm{of}\ \mathrm{the}\ \mathrm{cumulative}\ \mathrm{probability}\kern0.5em \mathrm{density}\kern-20.5em \mathrm{function}\kern0.22em \mathrm{of}\;\mathrm{the}\;\mathrm{normal}\;\mathrm{function}\end{array} $$

3. Each paired trial with a pull-force slope in the interval [*t*_CSbreak_, UUR] less or more than the confidence band CB_0.95_ is classified either as with cued force change (with CFC) or as without cued force change (without CFC).

*t*_break_ is calculated using Yorick language [36]. All other statistical computations were carried out using the language for statistical computing “R” [37].

### Dynamic Torque Analysis

**Fig. 1:**
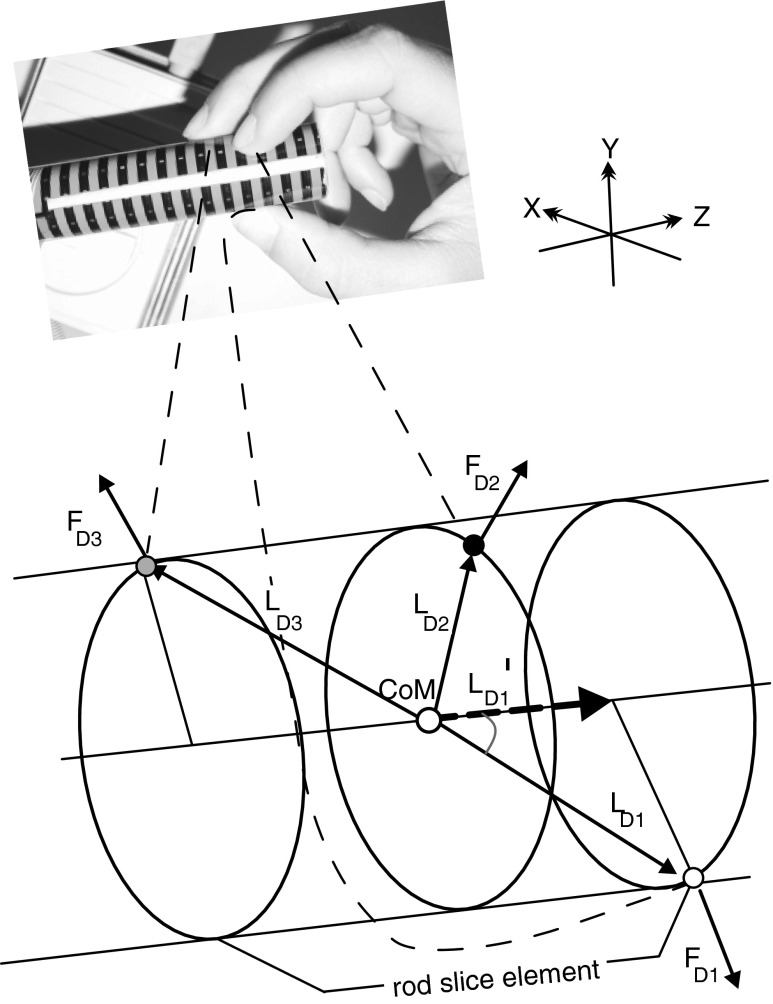
Finger position and torque calculation for the raspberry task. The *inset* shows the grip rod and the attached force-measurement film as well as the fingers with their position during the raspberry task. The rod slice element is part of the rod between the remotest fingers (here: *D1* and *D3*). *D1*: thumb, *D2*: index finger, *D3*: middle finger. *F*_*D1*–*3*_ force vectors given by fingers *D*_*1–3*_, *L*_*D1–3*_ lever vectors given by fingers *D*_*1–3*_, *L*_*D1*_’ (*dashed vector*) is the effective length of the lever of *L*_*D1*_; CoM: centre of mass

A detailed description of the dynamic torque analysis has been given elsewhere [26, 27]. Grip forces of individual fingers were measured as well as the pull force representing the sum of all tangential forces. Individual fingers exert orthogonal forces on the contact surface of the grip rod (Fig. 1, *F*_*D1*_, *F*_*D2*_, *F*_*D3*_). The positions of fingers on the surface are not predetermined but were unrestrained and freely selectable according to the individual geometric and physiological properties of the subject’s hand. In this study, we will use “torque” to designate a force moment resulting from orthogonal finger forces which would tend to deviate the rod from the pull-direction and represent losses that subjects try unconsciously to minimise.

Individual grip forces and finger positions were derived from the force-measuring film data as described above (section “Force-Measuring Film and Grip Rod”). From the finger positions, a gripped rod slice element and its centre of mass (CoM) were defined. The rod slice element is a virtual part of the rod between the remotest fingers (e.g. rod part between thumb (D1) and middle finger (D3) in Fig. 1). Levers were derived from the position of individual fingers and the centre of mass of the rod slice element. Individual torques were derived from the vector product of finger force and the lever from the position on the rod, where the force is exerted to the centre of mass of the rod slice element.

For a comparison of the effect of factor “lever” and factor “grip force” on torque, the average torque, average total grip force and the average of the effective lever length were calculated for the following points in time: *start* = from *t*_start_ to 500 ms afterwards, *mid*-*1* and *mid*-*2* = data within 25–75 % (middle half) of the interval length of interval-1 and interval-2, respectively (see section “Analysis of Pull-Force Slope Change”), *int*_break_ = from 250 ms before *t*_break_ to 250 ms after, and *ref* = 500 ms before CS until CS as reference interval. Total grip force was calculated as the sum of absolute values of individual finger forces. Total effective lever length was calculated under the following considerations. Given that grip forces are orthogonal forces to the contact surface of the grip rod and, hence, are orthogonal to the z-axis and, given that force vector *F*_*i*_ and lever vector *L*_*i*_ are not orthogonal to each other (e.g. *F*_*D1*_ and *L*_*D1*_ in Fig. 1), the torque produced by vector *F*_*i*_ on the centre of mass is not exerted along the full length of vector *L*_*i*_ but, according to vector algebra, along a shorter lever (*L*_*i*_’). The effective length of lever *L*_*i*_’ is calculated as the length of the projection of *L*_*i*_ on the z-axis (pull-axis, e.g. *L*_*D1*_’ in Fig. 1). In cases in which a finger is positioned directly above the centre of mass (e.g. *F*_*D2*_ in Fig. 1), the effective lever becomes zero and indeed a finger in this position does not contribute to a torque responsible for a virtual deviation out of the pull-axis. The total effective lever length is given by the sum of the absolute values of the lengths of all projected levers. For convenience, total grip force and total effective lever length are expressed in the following as grip force and lever, respectively.

## Results

A recent study showed that anticipatory force changes could be cued in young subjects [15]. The aim of the present study was to analyse the influence of the cerebellum in cueing grip force and pull-force changes. The two force changes were analysed in an isometric precision task in which a specialised grip rod had to be gripped. Then, a smoothly and linearly increasing pull force had to be generated against the locked rod (pull phase) until the unpredictable unlocking of the rod (UUR) occurred. After UUR, the rod began to move, participants had to stop the movement with as little effort as possible (pluck phase). The transition from the pull to the pluck phase required changes from increasing grip force and pull force to an immediate and rapid decrease of these forces. Hence, in this task, the UUR—acting as an unconditioned stimulus in protective reflex conditioning, e.g. eye blink conditioning, for review see [29]—provokes an unpredictable force change. During paired trials, an auditory signal (1 kHz, 75 dB) was given as cueing stimulus (CS) 465 ms before UUR terminated at UUR. Significant changes in grip forces and/or pull force within the CS-UUR window were judged as cued. Eleven patients representing with degenerative cerebellar disease (7 females, 4 males, age 51.0 ± 12.6 years) and 11 healthy, sex-, age-, and handedness-matched control subjects (age 50.7 ± 12.9 years) participated in the study (Table 1).

### Cued Force Changes

**Fig. 2:**
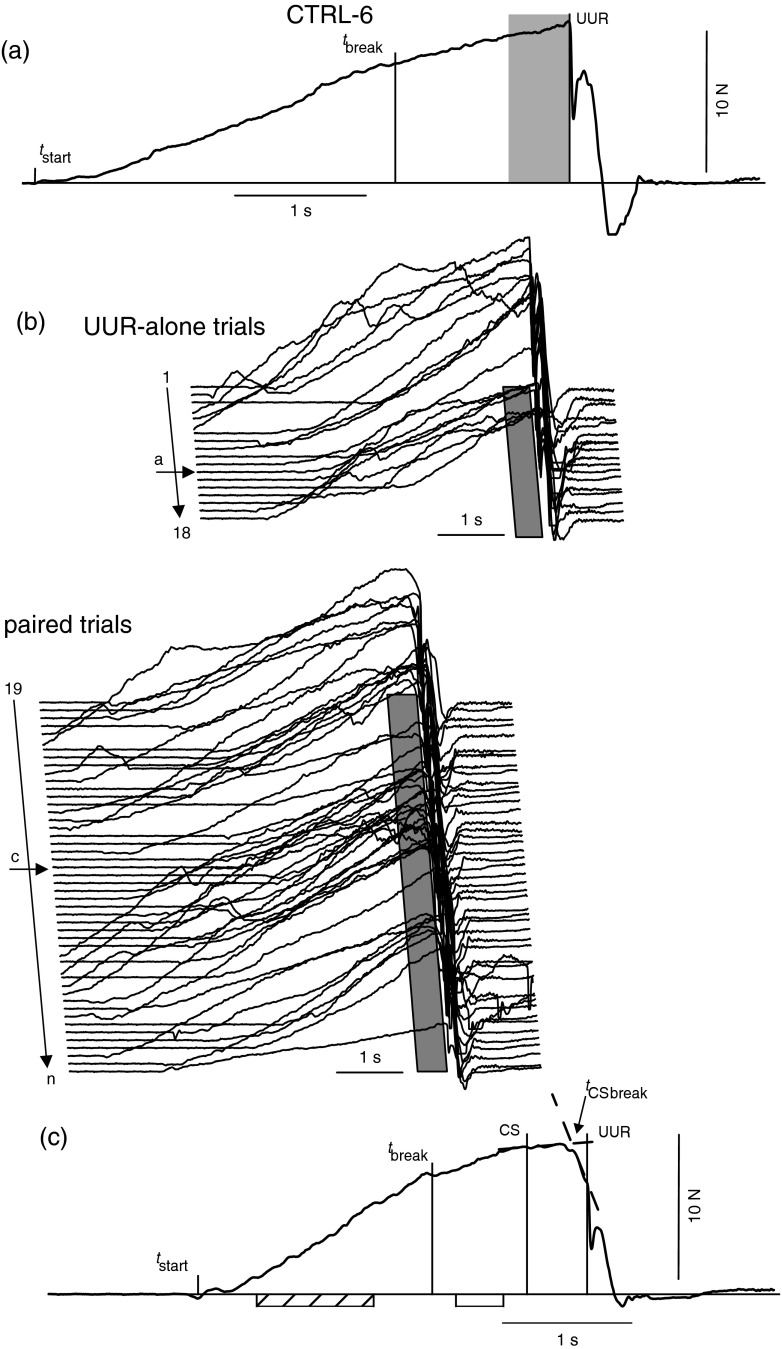
Characteristic pull force traces of a single subject (CTRL-6, Table 1). **a** Characteristic UUR-alone trial, *t*_start_: start of the movement, *t*_break_: time of pull-force slope change, UUR: unpredictable unlocking of the rod. **b** Individual pull force trials performed sequentially. UUR-alone trials are shown in the *upper* part and paired trials are shown *below*. The data are aligned to UUR. The CS-UUR window in paired trials and the equivalent interval in UUR-alone trials is given by a *grey rectangular below* the trials. *Arrows* with the notations *a, c* indicate single trials shown in (**a**) and (**c**). **c** Characteristic paired trial, CS: cueing stimulus, *t*_CSbreak_: time of pull-force slope change in the CS-UUR window characterising a cued force change (CFC). *Hatched* and *white bars below* the x-axis indicate the middle half of interval-1 and interval-2, respectively

**Fig. 3:**
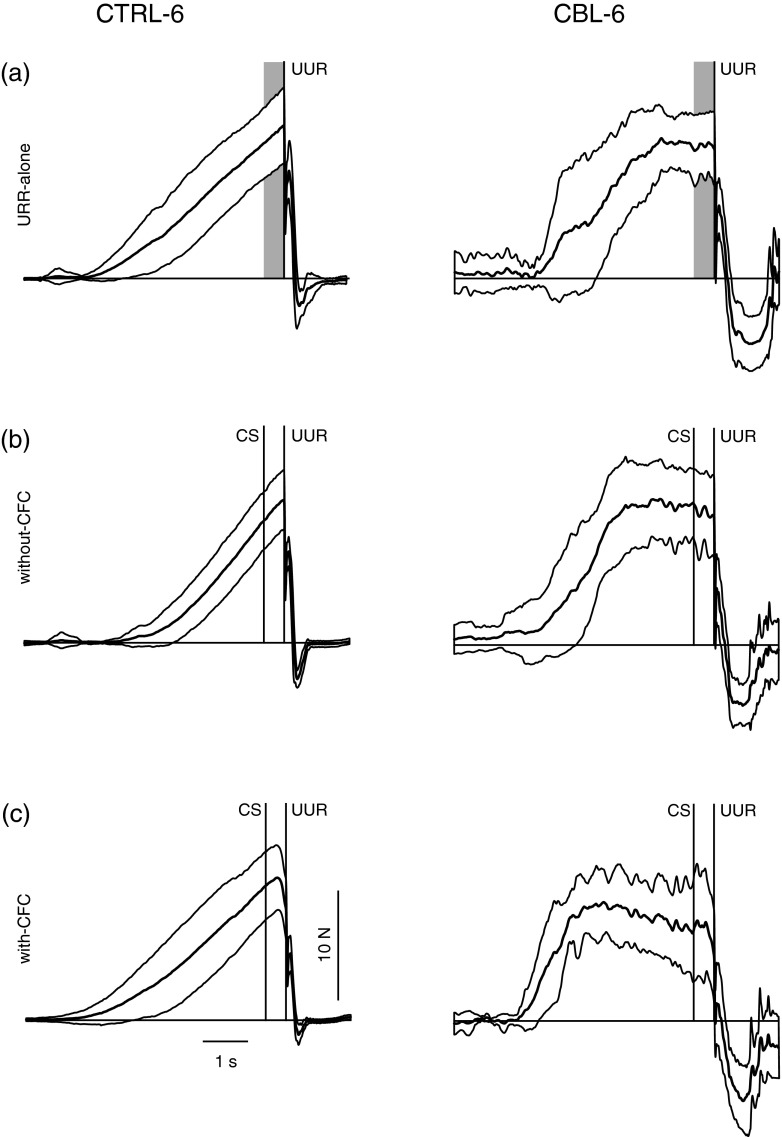
Characteristic averaged pull forces of two subjects (CTRL-6 and CBL-6, Table 1) for UUR-alone trials (**a**), and for paired trials without CFC (**b**), and those with CFC (**c**). **a**–**c** Averaged pull force ± SD, data were aligned to the UUR at *t* = 0. **a** *Grey rectangular* indicates the equivalent interval of the CS-UUR window in paired trials

A main characteristic of the raspberry task is that any changes of the pull force reflect changes in grip forces in any of the fingers applying in the task. Hence, analysis of pull-force changes gives a comprehensive view of the underlying grip force changes. In general, all participants produced increasing pull forces and the pull force slope was reduced after *t*_break_ (e.g. Fig. 2a,c). Hence, this time point divided the trial into two intervals: interval-1 from *t*_start_ to *t*_break_ and interval-2 from *t*_break_ to CS in paired trial or the equivalent time point in UUR-alone trials. During paired trials, cued force changes (CFC) are visible as a second change in pull force (*t*_CSbreak_) in the CS-UUR window and can be determined using a two-linear-splines regression model (Fig. 2c, dashed line). Characteristic data of a single subject (CTRL-6, Table 1) are summarised in Fig. 2. In UUR-alone trials, pull force increased up to the UUR (Fig. 2b upper part). This was followed by a sequence of three changes: (i) an immediate break-down of the pull force as a result of the unlocked rod, followed by (ii) a rapid increase of pull force that is subsequently reduced afterwards (iii). In paired trials, pull force decreased frequently in the CS-UUR window (Fig. 2b lower part, e.g. Fig. 2c). This subject showed a significant change in the pull force approximately 340 ms after the CS in 60 % of paired trials. The comparison of the averaged pull force data obtained during paired trials with cued force change (with CFC, see section “Analysis of Pull-Force Slope Change”) with those obtained without cued force change (without CFC) showed a remarkable reduction of the pull force in trials with CFC within the CS-UUR window (Fig. 3b,c CTRL-6).

For comparison, the averaged pull force data ± SD from CBL-6 (the matched subject to CTRL-6) are shown in Fig. 3 also. This subject showed a characteristic pattern of pull-force slopes: initially a strong increase during interval-1 and afterwards a strong decrease in pull force (Fig. 3a–c, CBL-6). At group level, CBL produced a mean pull-force slope of 7.5 N/s (SEM: 0.2) during interval-1 and of 0.9 N/s during interval-2 (SEM: 0.1). Both slopes were significantly different (*t* test, *P* < 0.001) compared with CTRL (means ± SEM: 4.6 ± 0.1 N/s and 2.0 ± 0.1 N/s, respectively). Similar results were found for the UUR-alone trials (data not shown). In addition, during paired trials, (Fig. 3c, CBL-6) CBL-6 produced CFC to a lesser extent (20 % of paired trials).

**Fig. 4:**
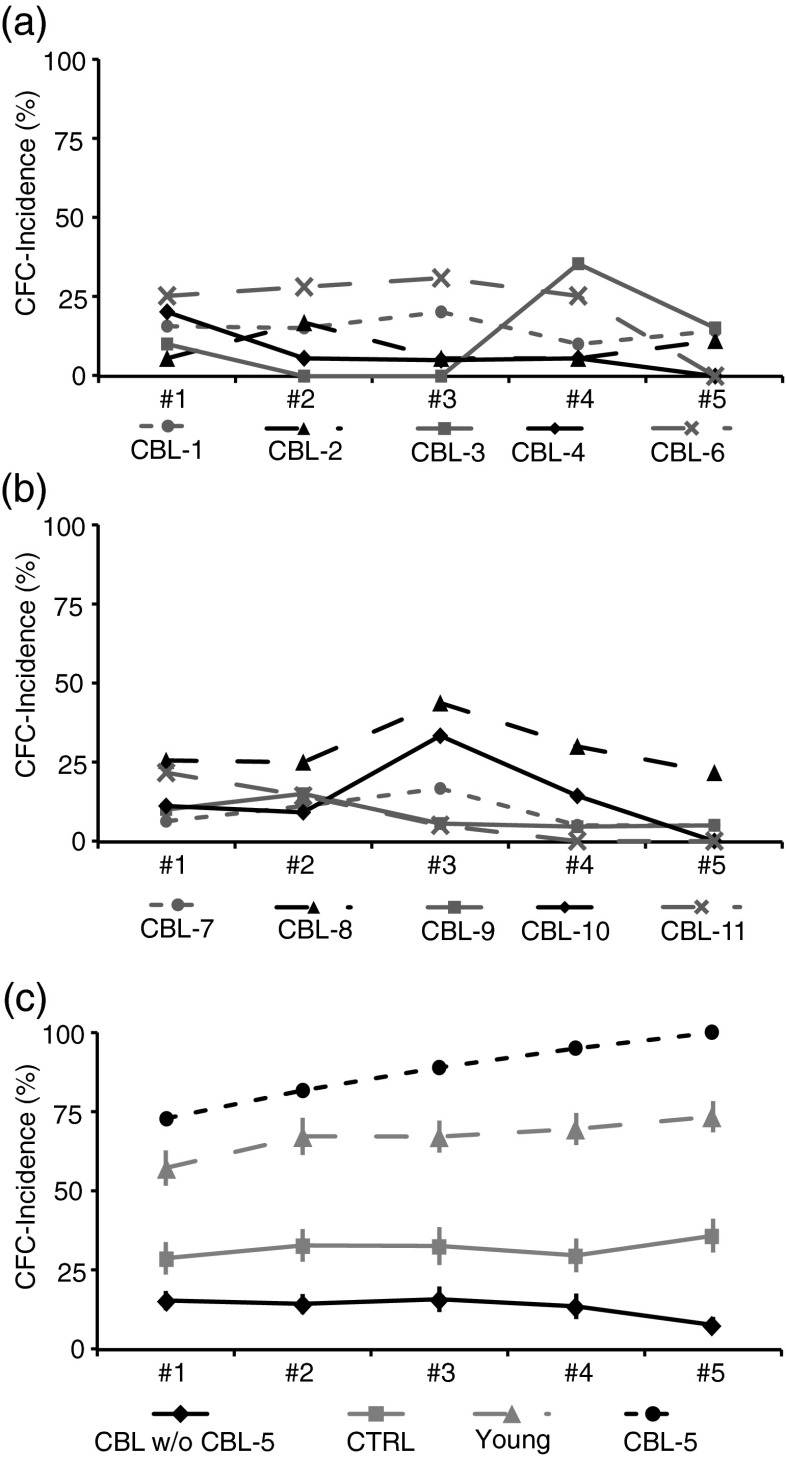
Variation of individual CFC incidence and group means. **a**–**c** CFC incidence calculated in blocks of ten trials (#1–#5) for all patients participating in this study (see Table 1). **c** Group means of CFC incidence ± SEM for CBL without CBL-5: *black solid line* with *diamonds*, CTRL: *grey solid line* with *squares*, Young (see [15]): *grey long*-*dashed line* with *triangles* and individual data from CBL-5: *black short*-*dashed line* with *dots*

The individual acquisition of CFC varied considerably within CBL as depicted in Fig. 4. The mean CFC incidence was approximately 13 % in paired trials (Fig. 4c black solid line with diamonds, range = [7–29 %]) with the exception of CBL-5 manifesting a CFC incidence of 87 % (Fig. 4c, black short-dashed line with dots). This CFC incidence was much higher than those found in CTRL (mean: 32 %; Fig. 4c, grey solid line with squares) and was also higher than those reported for a group of young subjects (Fig. 4c, Young, grey long-dashed line with triangles; see [15]). Hence, patient CBL-5 was excluded from the following analysis.

The overall group data showed that in CBL CFC appeared significantly less frequently as in CTRL (*t* test, *P* < 0.05). In line with this result, we noticed that the occurrence of the first CFC in CTRL occurred in median with the fourth presentation of the CS whereas in CBL with the eighth presentation. Analysing the data in blocks of ten trials, in CBL, the CFC incidence in the first block was 15 % and decreased to a minimum of 8 % in the fifth block (Fig. 4c, black solid line with diamonds). In contrast, CTRL had a CFC incidence in the first block of 27 % and reached its maximum of 35 % in the fifth block (Fig. 4c, grey solid line with squares). However, the change of CFC incidence in CTRL was not significant whereas the decrease in CBL was significant (*t* test one-sided, *P* < 0.05). Hence, patients showed a significant reduction of CFC generation.

### Adaptive Gripping Behaviour

**Fig. 5:**
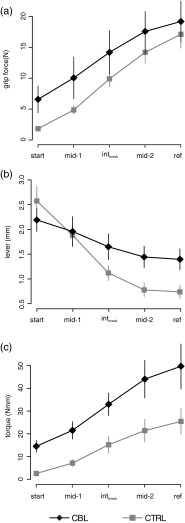
Adaptive changes of the gripping behaviour. Group means ± SEM of grip force (**a**), lever (**b**) and torque (**c**) for five epochs of a trial. *start* = from *t*_start_ to 500 ms afterwards, *mid*-*1* and *mid*-*2* = middle half of the interval length of interval-1 and interval-2 (see Fig. 1), *int*_break_ = from 250 ms before *t*_break_ to 250 ms after, and *ref* = 500 ms before CS until CS. **a**–**c** CBL: *black line* with *diamonds*, CTRL: *grey lines* with *squares*

Adaptive changes of the gripping behaviour during a trial were studied in five epochs applying the dynamic torque analysis (section “Dynamic Torque Analysis”). Levers and torques were derived from grip forces and geometric properties of the grip rod. The five epochs were characterised by *start* = from *t*_start_ to 500 ms afterwards, *mid*-*1* and *mid*-*2* = data within 25–75 % (middle half) of the interval length of interval-1 and interval-2 (Fig. 2c hatched bar and white bar under the x-axis, respectively), *int*_break_ = from 250 ms before *t*_break_ to 250 ms after, and *ref* = 500 ms before CS until CS. Group means (±SEM) are given in Fig. 5. Both groups increased grip force during the trial (Fig. 5a, CBL: black line with diamonds, CTRL: grey line with squares) which was necessary to increase pull force over time. The main difference was that CBL started with an approximately fourfold higher grip force than CTRL (6.6 N vs. 1.8 N, respectively) and the increase of grip force during the trial (comparing *ref* with *start*) was just threefold in the patient group whereas it was ninefold in the control group. The mean lever depends on the finger position and is a measure of how skilful the fingers are positioned on the rod. The mean levers were at *start* comparable in both groups (CBL: 2.2 mm vs CTRL: 2.6 mm). Both reduced the lever during the trial (Fig. 5b). The main difference was that CTRL reduced the lever more than CBL, the first showing the strongest reduction approximately at *t*_break_. Hence, the mean lever in epoch *ref* was 0.7 mm for CTRL and 1.4 mm for CBL. In consequence, the resulting torque was different between CBL and CTRL throughout the complete trial (Fig. 5c). The largest difference was seen at *start* with a relation of 6 times higher in CBL than in CTRL. Torque reached the maximum at *ref* with 49.7 Nmm in CBL and 25.5 Nmm in CTRL. In consequence, both groups adapted their grip during the trial. However, CBL resulted in a higher torque due to the higher grip forces from start on and a limited ability to reduce the lever.

## Discussion

In this study, we examined the involvement of the cerebellum in establishing cued force changes in the raspberry task. In the raspberry task, the cueing stimulus was associated with a complex change in motor behaviour—an anticipatory adjustment of forces which occurs during an ongoing movement already started. The results show that patients presenting with degenerating cerebellar disease were able to elicit CFC but with a lower incidence than in the control group. This observation strongly implies involvement of the cerebellum not only in the classical conditioning of protecting reflexes but also in the association of an auditory stimulus with a complex motor behaviour. It should be noted, however, that there was large between-subject variability with regard to CFC incidence within CBL (Fig. 4). In particular, one patient (CBL-5) produced with a mean incidence of 87 % a CFC incidence which was higher than that observed in CTRL. Nevertheless, this subject did not differ in any of the clinical scores to those of other patients in this group (Table 1). Comparison of the CFC incidence of this study with other studies analysing the conditioned response incidence (CR incidence) in reflex tasks (Table 2) shows that in reflex tasks CTRL increases the CR incidence over repetition (Table 2 CTRL 1st block vs. 5th block) whereas CBL were hardly able to do so. This is also true for the CFC incidence in this study (Fig. 4c, CBL black solid line, CTRL grey solid line). Hence, CBL are able to elicit conditioned responses, but repetition brings very little improvement in performance. The only exception was the lower limb withdrawal reflex in standing subjects. In this special case [11, 12, 14, 38], CBL were able to increase significantly the CR incidence (Table 2 CBL 1st block vs. 5th block). Comparing the mean CFC incidence of CTRL (1/3) with the CFC incidence of young subjects 2/3, see [15], suggests that the capability to produce CFC depends primarily on the cerebellum, but an age-dependency has also to be taken into account. Age-dependency in conditioning reflexes has been studied extensively by Woodruff-Pack [39].

**Table 2:** Comparison of the CFC incidence in this study with the CR incidence of other studies

Task	Study		Percent incidence			
			CBL		CTRL	
			1st block	5th block	1st block	5th block
Eye-blink reflex	Gerwig et al. (2010), [38]		9	10	5	22
Lower limb withdrawal reflex	Reclining	Timmann et al. (2000), [7]	9	2	20	46
	Standing	Kutz et al. (2014)^a^, [11]	10	25	32	65
Postural reflex	Kolb et al. (2004), [13]		2	3	0	20
Raspberry task	This study		15	8	27	35

^a^The CR incidence in this study was calculated in block of nine trials. The value given here is the mean of the 5th and the 6th blocks

In the raspberry task, the positions of fingers are unrestrained and freely selectable according to the individual geometric and physiological properties of the subject’s hand [28]. From the individual grip forces, the positions of the fingers, and the geometric properties of the grip rod torques that would deviate the rod from the pull-direction could be calculated. These torques represent losses that subjects minimise unconsciously. We also studied the involvement of the cerebellum in minimising these losses. Both groups showed an increase of the torque during the ongoing trial. A detailed analysis revealed that the factor lever showed the largest difference between the groups. The lever is a comprehensive measurement of subjects grip behaviour. For a reduction of the lever, a greater skill is needed. Both groups started the trial with a similar lever (>2 mm), and both groups were able to reduce the lever while the ongoing trial, CBL reduced the lever to 64 % of its initial value whereas CTRL reduced it to 29 %, showing that the ability of CBL to optimise their grip is compromised. Re-positioning the fingers needs adequate activation of the muscle of the interphalangeal joints. CBL are known to be restricted in multi-joint movements of the arm and shoulder joints, e.g. [40–42]. It is assumed that this restriction is caused by an inaccurate prediction of sensory information during self-generated movements by the cerebellum [43, 44]. So that one explanation for the reduced re-positioning found in CBL would be poor prediction of the sensory information.

A further explanation is related to the innervations pattern of finger muscles. Anatomical tracing studies in primates showed that the primary motor cortex terminates mono-synaptically [45] as well as di-synaptically [46] on motor neuron pools of finger muscles demonstrating a complex matrix of descending connections, for review see [47]. These connections facilitate both contralateral extensor and flexor muscles, for review see [48], and allow precise and gradual positioning of fingers. Recent data provide evidence that in addition to the cortico-spinal tract, the reticulo-spinal tract exerts influence over hand movements in primates [48] and in humans [49]. In humans, the function seems to be limited to coordinated movement of the whole hand [49], most probably because the reticulo-spinal tract tends to facilitate flexors and suppress extensors of the hand in use [50, 51]. The reticulo-spinal tract is strongly innervated by the cerebellum [52–55]. The importance of this tract has been demonstrated recently in a study in which electrical stimulation of human cerebellar hemispheres elicited finger movements [56], e.g. Fig. 2. Interestingly, the authors noted that the latency for eliciting finger muscle activation due to cerebellar stimulation were similar with results of electrical stimulations of the primary motor cortex in a control group [56], e.g. cerebellum 28 ms vs. primary motor cortex 26 ms. Hence, two separate and independent pathways must be assumed, both able to elicit finger muscle contraction. The results of this study suggest that an imbalance of the two pathways exist in our patients. This has the effect that in the patients the cerebellar degeneration results in a small but significant over-excitation of the cerebello-reticulo-spinal tract compared with the cortico-spinal tract. This would have the effect that stronger activation of finger flexors would reduce the ability for re-positioning the fingers—a process requiring extensor activation—as seen in our study. It is noteworthy that the reduction of grip force inside the CS-UUR window—which is necessary to react to on the forthcoming unlocking of the rod—is severely limited in the patient group, since activation of the finger extensors is also necessary here. In short, the putative imbalance between the two pathways would explain in part the lower incidence of a CFC in CBL compared with that of CTRL.
